# Adherence to zinc supplementation guidelines for the treatment of diarrhea among children under–five in Uttar Pradesh, India

**DOI:** 10.7189/jogh.05.020410

**Published:** 2015-12

**Authors:** Laura M Lamberti, Christa L Fischer Walker, Sunita Taneja, Sarmila Mazumder, Robert E Black

**Affiliations:** 1 Johns Hopkins University Bloomberg School of Public Health, Department of International Health, Baltimore, MD, USA; 2 Centre for Health Research and Development, Society for Applied Studies, New Delhi, India

## Abstract

**Background:**

There is limited evidence on adherence to the recommended dose and duration of zinc supplementation for diarrheal episodes in children under five years of age. In selected districts of Uttar Pradesh, India, we sought to assess adherence to the nationally advised zinc treatment regimen (ie, 10 mg/day for ages 2–6 months and 20 mg/day for ages 7–59 months for 14 days) among caregivers of zinc–prescribed children.

**Methods:**

We identified and conducted follow–up visits to children advised zinc for the treatment of diarrhea. At the initial visit, we collected data on the treatment instructions received from providers. Caregivers were asked to record treatments administered on a pictorial tracking form and were asked to retain all packaging for collection at follow–up. We quantified the average dose and duration of zinc therapy and built logistic regression models to assess the factors associated with caregiver adherence to national guidelines.

**Results:**

Caregivers administered zinc for an average of 10.7 days (standard deviation (SD) = 3.9 days; median = 13 days), and 47.8% continued treatment for the complete 14 days. Among children receiving zinc syrups and tablets respectively, the age appropriate dose was received by 30.8% and 67.3%. Adherence to age appropriate dose and continuation of zinc for 14 days were highly associated with having received appropriate provider instructions.

**Conclusions:**

Our results indicate moderate–to–good adherence to national zinc treatment guidelines for diarrhea among caregivers in rural India. Our findings also highlight the importance of provider guidance in ensuring adherence to zinc dose and duration. Programs aiming to scale–up zinc treatment for childhood diarrhea should train providers to successfully communicate dosing instructions to caregivers, while also addressing the tendency of caregivers to terminate treatment once a child appears to have recovered from an acute diarrheal episode.

The efficacy and effectiveness of therapeutic zinc supplementation in reducing the duration and severity of diarrhea among children under five years of age has been well–documented [1-3]. In response to mounting evidence, UNICEF and WHO revised the global childhood diarrhea treatment guidelines in 2004 to include continued feeding, low–osmolarity oral rehydration salts (ORS), and daily zinc supplementation for 10–14 days with 20 mg/d for children aged 6–59 months and 10 mg/d for infants aged <6 months [4]. Despite the global recommendation and the incorporation of zinc into the national diarrhea treatment policies of a growing number of countries, zinc treatment has failed to become available at scale over the past decade and attaining improved coverage of zinc, as well as ORS, has remained a challenge in most low– and middle–income countries [5].

In addition to concerns regarding access to zinc treatment for diarrhea among children under–five years of age, studies assessing adherence to the advised dose and duration of supplementation have called into question the quality of the regimen received by those actually treated with zinc [6-14]. A cluster randomized controlled trial (cRCT) in Bangladesh reported that on average children with diarrhea residing in intervention villages received only 7 days of the total 14–day zinc dosage [7]. In a study drawing evidence from cRCTs conducted in Brazil, Ethiopia, Egypt, Philippines and India (Nagpur and Lucknow), adherence to zinc for ≥10 days was 83.8% [6]. A cRCT conducted in Haryana, India found that in intervention villages the proportion of zinc–treated children receiving the full 14–day dose decreased from 70% to 61.9% when assessed at three and six months post–intervention, respectively [8]. These three studies highlight the challenges associated with achieving and maintaining high levels of zinc treatment adherence even under controlled research settings.

Evidence from observational studies suggests that in practice adherence to the zinc treatment guidelines may be less common among both providers and caregivers. In a 2005 evaluation of the Scaling up zinc for young children (SUZY) project, only 55.8% adhered to the advised 10–day duration of treatment, and the average zinc–treated child received zinc for 7–8 days in total [10]. In communities in Mali in which a zinc scale–up project was underway, adherence to 14 days of therapeutic zinc was 64% and though compliance with dosage instructions was generally good (94%), one infant received more than the age appropriate daily dose of zinc [14]. A cross–sectional study of caregivers of children under–five in Kenya reported low compliance with the guidelines on both zinc treatment duration (38%) and dosage (55%), with 32% of caregivers administering more than the age appropriate daily zinc dose [13].

Though there are limited studies assessing the issue of adherence to therapeutic zinc supplementation for diarrhea, the existing evidence highlights important research questions that should be addressed as the global community pushes toward the goal of scaling–up adequate diarrhea treatment. Evaluations of newly implemented diarrhea treatment programs should therefore be designed to not only gauge zinc coverage but also the level of adherence to the zinc treatment protocol among both providers and caregivers of children under–five. While coverage surveys capture the proportion of children with diarrhea who receive any course of zinc, it is possible that very minimal zinc treatment regimens do not confer the same benefits as the advised course. Therefore, the level of zinc adherence may affect the extent to which increases in coverage translate into impact on diarrhea morbidity and mortality outcomes in the long–term. Furthermore, monitoring adherence to zinc dosage instructions is important, since ingesting more than the age appropriate daily dose may pose a risk for zinc toxicity.

In an effort to address the question of zinc treatment adherence among caregivers of zinc–prescribed children under–five in rural India, we nested a sub–study within the external evaluation of the Diarrhea Alleviation through Zinc and ORS Treatment (DAZT) project in Uttar Pradesh (UP), India. To our knowledge, this is the first observational study to assess adherence to therapeutic zinc supplementation in rural India.

## METHODS

### Overview of the DAZT project and zinc adherence sub–study

The DAZT project was conducted in selected districts of UP from 2011–2014 with the goal of scaling–up ORS and zinc treatment of diarrhea among children 2–59 months of age. A complete description of the intervention and evaluation results are published elsewhere [15]. In brief, ORS and zinc supplies were made available to facility– and community–based public sector providers who also received thorough training in adequate treatment of childhood diarrhea. In the private sector, representatives of pharmaceutical companies were employed to solicit the sale of ORS and zinc to both qualified providers and informal, unlicensed providers.

The zinc adherence sub–study was designed to address the dearth of information on zinc treatment adherence among caregivers in rural India. Our main objective was three–fold: to estimate the proportion of caregivers who received zinc dose and duration instructions in agreement with national childhood diarrhea treatment guidelines; to estimate the proportion of caregivers that adhered to the course of zinc therapy advised by their child’s provider; and to quantify the average zinc treatment dose and duration received by children in this population. The study was conducted from May through July, 2014 in the Lucknow and Mohanlal Ganj tehsils of Lucknow district and the Badaun and Bisauli tehsils of Badaun district in UP.

### Sample size

In the absence of published estimates of zinc adherence in rural India, we employed the most conservative methodology and assumed the true proportion of adherent zinc–treated children to be 50%; this approach maximizes the sample size requirements and was the most appropriate given our limited knowledge on the outcome of interest in this population. We used Stata 12.0 statistical software to calculate the sample size required to generate a precision estimate of the point prevalence of adherent zinc–treated children within 10 percentage points with 95% confidence [16]. The resulting sample size requirement of 97 zinc–prescribed children was inflated to 120 to account for the possibility of loss–to–follow–up. We aimed to equally divide the required sample size of 120 zinc–prescribed children across the four included tehsils (ie, 30 per tehsil) but if interviewers encountered difficulty identifying zinc–prescribed children in a given tehsil due to unforeseen zinc treatment stock–outs or lower than anticipated prescribing practices, we allowed the sample size to be made up in the remaining tehsils.

### Data collection

In each tehsil, we randomly selected 12 rural villages for inclusion in the study using probability proportional to size (PPS) sampling. Trained interviewers visited all households within the selected villages to identify children meeting the following inclusion criteria: 1) aged 2–59 months; 2) episode of diarrhea (defined as ≥3 loose or watery stools in a 24–hour period) in the 7 days preceding the household visit; 3) receipt of zinc treatment for diarrhea in the 3 days preceding the visit. To ensure the accuracy of zinc reporting, interviewers verified the third enrollment criterion by asking to see any available treatments or packaging from treatments administered to the child. If more than one child in the 2–59 month age range resided in the household, the primary caregiver was instructed to base her responses on the youngest. Consenting primary caregivers of zinc–treated children were formally interviewed regarding the place of zinc procurement, zinc treatment instructions received, and the dose/duration of treatment to date.

Interviewers scheduled follow–up visits for 14 days after the initial visit to all households in which a child met the inclusion criteria. The caregiver was asked to retain the packaging from any treatments administered to the child during this period and was also shown how to use a pictorial tracking form to record days on which the child experienced diarrhea and days on which the child was administered ORS and/or zinc syrup or tablets. The tracking form also included slots in which to record common daily activities (ie, feeding and bathing) such that dummy variables could be generated to assess caregivers’ understanding of the tracking process. To reduce the threat of caregivers modifying their adherence behavior due to the scheduled follow–up visit, they were told that the purpose of the tracking form and visit was to check on the child’s well–being following the diarrheal episode.

During the follow–up visit, the interviewer administered questions on the child’s diarrheal episode and treatments given during the preceding 14 days and confirmed the caregiver’s responses by referring to the tracking form and all reserved packaging. Interviewers also questioned caregivers regarding diarrhea treatment preferences, perceived benefits of zinc and reasons for discontinuing zinc treatment.

### Statistical analyses

We conducted statistical data analyses using Stata 12.0 software [16]. We summarized the sociodemographic, diarrheal episode and zinc treatment characteristics of all children by calculating the means, standard deviations and medians of continuous variables and the proportions of categorical variables. We calculated the proportion of caregivers that received provider instructions on zinc dose and duration in agreement with the recommendation issued by the Government of India (GoI) and the Indian Academy of Pediatrics (IAP) (ie, 14 days supplementation with 10 mg/d for ages 2–6 months and 20 mg/d for ages 7–59 months) [17,18]; these guidelines differ slightly from WHO/UNICEF in the age cut–offs for dose but were the most appropriate gauge of adherence since DAZT project providers were trained according to the national protocol. We also calculated the proportion of caregivers that adhered to the provider–advised course of zinc therapy, the average duration of zinc treatment, and the proportion of children receiving an age appropriate dose. We conducted *z*–tests to assess the statistical equivalence of adherence by age category and zinc product formulation (ie, tablet or syrup).

We built three logistic regression models to assess the factors associated with adherence to the GoI/IAP advised dose and duration of zinc therapy with receipt of appropriate provider instructions as the primary explanatory variable. In each of the three models, respectively, we regressed: the log odds of continuing zinc for the complete 14 days onto an indicator of whether the provider advised zinc for 14 days (**model 1**); the log odds of receiving the age appropriate zinc dose onto an indicator of whether the provider gave such instructions (**model 2**); the log odds of receiving the age appropriate dose for 14 days onto an indicator of whether the provider advised the correct dose and duration of therapy. All models controlled for age category (7–59 months vs 2–6 months), duration of the diarrheal episode in number of days, caregiver education (at least 1 year of school vs no school), and poor socioeconomic status as indicated by the possession of a BPL card (**Table 1**). To adjust for correlation in adherence behavior at the tehsil level, we employed the robust cluster estimator of variance in Stata 12.0 [16].

**Table 1:** Sociodemographic and diarrheal episode characteristics of children with completed follow-up (n = 113)

	Number (%)
**Age of child (in months):**	
Aged 2–6 months	23 (20.3)
Aged 7–59 months	90 (79.7)
Mean±SD	17.7 ± 13.9
Median (range)	13 (2–59)
**Sex:**	
Male	48 (42.5)
Female	65 (57.5)
**Episode characteristics:**	
Blood in stool	13 (11.5)
Fever	83 (73.5)
Vomiting	40 (35.4)
Lethargic/irritable	84 (74.3)
Sunken eyes	54 (47.8)
Dehydration/*Pani ki kami*	71 (62.8)
Duration (days, mean±SD)	4.4 ± 3.0
Median (range)	3.0 (1–15)
**Ethnic group:**	
Scheduled caste	66 (58.4)
Scheduled tribe	2 (1.8)
Other backward castes	36 (31.9)
General	9 (8.0)
**Caregiver years of schooling:**	
Never attended school	44 (39.0)
Mean±SD	4.8 ± 4.5
Median (range)	5 (0–15)
Socioeconomic indicators:*	
Family possesses an APL card	47 (41.6)
Family possesses a BPL card	25 (22.1)
Family possesses an Antyodaya card†	13 (11.5)

SD – standard deviation, APL – above poverty line, BPL – below poverty line

*Government of India–issued ration cards for subsidized food and fuel.

†Antyodaya cards are issued to the poorest BPL families with income <250 rupees per month [18].

## RESULTS

**Figure 1:**
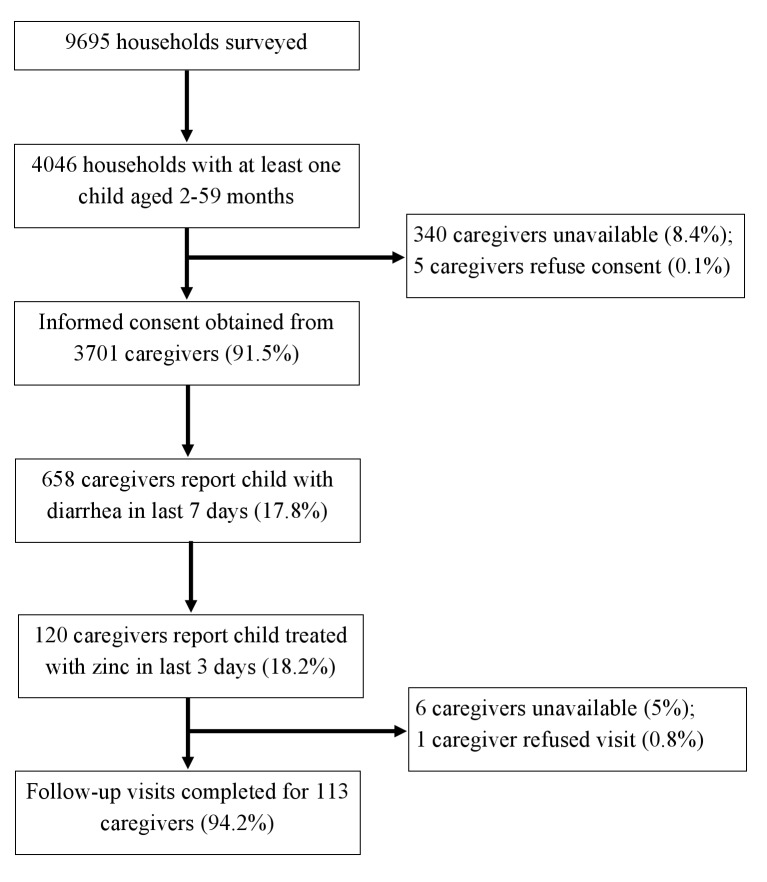
Data collection profile.

Follow–up visits were completed for 113 (94.2%) of the 120 caregivers of children meeting the inclusion criteria (**Figure 1**). The sociodemographic and diarrheal episode characteristics of these children are described in **Table 1**.

### Details of zinc treatment

All caregivers were in possession of the zinc supplements used at the initial household visit. The majority of zinc was procured through the public sector (88.5%) and specifically from a community–based provider cadre known as Accredited Social Health Activists (ASHAs) (85.8%; **Table 2**). All product procured through the public sector was obtained free of charge, whereas private sector zinc product was purchased. Zinc sulfate was the most commonly used product (90.2%) and tablets were the most common formulation (89.3%; **Table 2**). All zinc products obtained from the public sector were in tablet form, and all but one zinc course procured from the private sector were syrups.

**Table 2:** Description of zinc product and place of procurement (n = 113)

	Number (%)
Place of zinc procurement:*	
Public sector place of procurement:	100 (88.5)
– Accredited social health activist (ASHA)	97 (85.8)
– Anganwadi worker or center	3 (2.7)
Private sector place of procurement:	14 (12.4)
– Private provider	9 (8.0)
– Chemist	5 (4.4)
**Zinc product:**	
Zinc sulfate	102 (90.2)
Zinc acetate	8 (7.1)
Zinc gluconate	3 (2.7)
**Zinc formulation:†**	
Syrup	13 (11.5)
Tablets	101 (89.3)

*In one case, zinc was procured from both the public and private sectors.

†In one case, zinc was obtained in both tablet and syrup formulations. All zinc products obtained via the public sector were in tablet form; all but one zinc product procured from the private sector was in syrup form.

### Reported zinc treatment instructions

Only a small proportion of caregivers (3.5%) received a zinc instructional pamphlet from the provider who advised treatment, but the majority reported receiving provider instructions on how to prepare and administer zinc (96.5%), on how long to continue the zinc treatment course (90.3%), and on the daily dose to administer (100%). On average, caregivers were advised to give zinc for 13.8 days (SD = 1.2 days); 89.4% were told to continue treatment for 10–14 days and 85.8% for exactly 14 days. Among children 2–6 months of age, appropriate dosage instructions (ie, 1/2 tablet/d or 5 mL/d) were received by 65% and 33% of those treated with tablets and syrups, respectively. In the older age group, appropriate dosage instructions (ie, 1 tablet/d or 10mL/d) were received by 69% treated with tablets and 20% treated with syrups. In total, 55.8% (n = 63) of caregivers were advised according to the GoI/IAP guidelines on both dose and duration of zinc treatment [17,18]. In addition to zinc, 76.1% (n = 86) were also advised to administer ORS.

### Reported zinc treatment adherence

On average, children received zinc for 10.7 days (SD = 3.9 days, median = 13 days). Zinc treatment was continued for 10–14 days and for the complete 14 days by 63.7% (n = 72) and 47.8% (n = 54) of caregivers, respectively. The age appropriate dose was administered to 30.8% of the 13 syrup–treated children and to 67.3% of the 101 tablet–treated children.

Of the 97 caregivers instructed by providers to continue zinc therapy for 14 days, 52.6% adhered. There was no statistically significant difference in adherence to the duration of treatment by the child’s age (*P* = 0.996). Adherence to the advised 14 days was higher among children treated with tablets (53.3%) compared to syrups (40.0%) but this difference was not statistically significant (*P* = 0.563).

Adherence to dosage instructions was 87.5% among the 72 caregivers who received age appropriate advice; when stratified by age, 92.9% of caregivers of children ≤6months were dose–adherent compared to 86.2% in the older age group (*P* = 0.500). There was no statistically significant difference in adherence to age appropriate dosage instructions by product formulation of tablets (75.0%) compared to syrups (88.2%, *P* = 0.437). No child received more than one zinc dose per day but, but 2 (20.0%) syrup–treated and 4 (66.7%) tablet–treated children aged 2–6 months received the daily dose intended for children in the older age category.

Of the 63 caregivers advised appropriately on both dose and duration, 46.0% (n = 29) complied with both sets of instructions. All 86 caregivers told to administer ORS did so at least once during the episode.

### Factors associated with zinc treatment adherence

Continuation of zinc treatment for 14 days and adherence to age appropriate dose were highly associated with appropriate provider instructions (**Table 3**). Controlling for age and other factors, the odds of continuing treatment for 14 days (adjusted odds ratio, aOR = 6.43; 95% confidence interval (CI) = 3.09–13.37) and of administering the correct dose for age (aOR = 32.46, 95% CI 8.06–130.66) were elevated among caregivers instructed accordingly. In addition, the odds of adhering to the GoI/IAP guidelines on both dose and duration were higher among caregivers who received such advice from providers (aOR = 9.97, 95% CI 4.10–24.25). Age category, episode duration, caregiver education and household BPL status were not associated with adherence to guidelines on dose and duration when assessed as separate outcomes (**Table 3**; **Models 1 and 2**), but adherence to both dose and duration was lower among caregivers of children aged 7–59 months (aOR = 0.44, 95% CI 0.22–0.87) and those from households below the poverty line (aOR = 0.45, 95% CI 0.22–0.92) (**Model 3**).

**Table 3:** Factors associated with adherence to Government of India and the Indian Academy of Pediatrics guidelines on the dose and duration of zinc therapy for diarrhea*

	Model 1	Model 2	Model 3
**Outcome**	**Zinc continued for 14 days**	**Age appropriate zinc dose**	**Age appropriate zinc dose for 14 days**
	Adjusted odds ratio (95% confidence interval)		
Appropriate provider instruction†	6.43 (3.09–13.37)‡	32.46 (8.06–130.66) ‡	9.97 (4.10–24.25) ‡
Child age:			
7–59 months	1.44 (0.65–3.18)	0.29 (0.03–2.54)	0.44 (0.22–0.87)§
2–6 months	1.0	1.0	1.0
Episode duration (days)	1.07 (0.99–1.14)	1.00 (0.82–1.22)	1.05 (0.92–1.21)
Caregiver education:			
≥1 year schooling	1.07 (0.77–1.50)	1.08 (0.47–2.47)	0.86 (0.50–1.48)
Never attended school	1.0	1.0	1.0
Household below poverty line¶	1.01 (0.39–2.58)	0.57 (0.24–1.39)	0.45 (0.22–0.92)§

*Government of India/India Academy of Pediatrics guidelines advise zinc supplementation for 14 days in the daily tablet/syrup dose of 10 mg/5mL for infants aged 2–6 months and 20 mg/10 mL for children aged 7–59 months.

†Model 1: provider advised zinc for 14 days; Model 2: provider advised zinc dose appropriate for child’s age; Model 3: provider advised age appropriate dose to be continued for 14 days.

‡Statistically significant: *P* < 0.001.

§Statistically significant: *P* < 0.05.

¶As indicated by possession of a Below Poverty Line card.

The majority of caregivers who failed to administer zinc for the full 14 days reported the child’s recovery from diarrhea as the main reason for discontinuing zinc therapy (69.5%; **Table 4**). Commonly reported reasons also included administering another treatment (27.1%) and the perception that zinc was not working (17.0%). Running out of zinc product (5.1%) and the inability to afford zinc (3.4%) were mentioned by only a small proportion of caregivers, and concerns over vomiting or dislike of taste were not reported at all.

**Table 4:** Reported reasons for shortened duration of treatment among caregivers who administered zinc for <14 days (n=59)

	Number (%)
Reported reason zinc given for <14 days*	
Gave zinc for the advised number of days†	4 (6.8)
Child recovered	41 (69.5)
Child was given other treatment	16 (27.1)
Zinc was not working	10 (17.0)
Ran out of zinc supplies	3 (5.1)
Could not afford more zinc supplies	2 (3.4)
Child vomited	0
Child did not like taste of zinc	0

*Column totals exceed 59 as more than one reported reason was permitted.

†Comparison of the advised duration of treatment as reported during the initial visit to the total number of days zinc was given as assessed at follow–up shows the 4 respondents who said they gave zinc for the recommended number of days actually continued treatment for less than the advised number of days.

### Caregiver perceptions of zinc treatment

Zinc was not frequently reported as the preferred treatment for childhood diarrhea (29.2%) compared to ORS (58.4%), syrups (81.4%) and tablets (92%). The perceived benefits of zinc among caregivers included reduced stool frequency (56.6%) and volume (5.3%), as well as decreased duration (18.6%) and severity (5.3%) (**Table 5**). Caregivers also reported that zinc is good for diarrhea (47.8%) and makes children healthier and stronger (40.7%). However, 15.9% of caregivers were unable to list any benefit of zinc.

**Table 5:** Reported benefits of zinc among enrolled caregivers (n=113)

	Number (%)*
Reduces frequency of stool	64 (56.6)
Good for diarrhea/acts as drug for diarrhea	54 (47.8)
Makes child stronger/healthier	46 (40.7)
Treats/reduces risk of disease or illness	46 (40.7)
Reduces duration of diarrhea	21 (18.6)
Reduces severity of diarrhea	6 (5.3)
Reduces stool volume	6 (5.3)
Acts as a tonic after diarrhea	5 (4.4)
No benefit reported	18 (15.9)

*Column totals exceed 113 as more than one response was permitted.

## DISCUSSION

Our study sheds light on provider and caregiver adherence to the national guidelines on zinc treatment for childhood diarrhea in rural India. The overall results are encouraging, illustrating moderate–to–good adherence to the GoI/IAP zinc therapy protocol by providers and to provider instructions by caregivers.

Among providers, the proportion advising continuation of zinc for 14 days (85.8%) was higher than that advising age appropriate dose (63.7%). In addition, a higher proportion of providers offered correct dosage instructions on tablets than syrups. We observed the opposite trend among caregivers for whom adherence to provider instructions on the 14–day duration of zinc treatment (52.6%) was lower than those on age appropriate dose (87.5%). This finding suggests that the zinc adherence challenges are different for providers and caregivers and thus critical to future program planning. Providers may experience difficulty in recalling and/or communicating zinc dose instructions, which are complicated by age cut–offs and differences by product formulation (ie, tablet vs syrup). On the other hand, caregivers are required to remember only one set of dosage instructions tailored to their child’s specific age and product formulation and are perhaps less likely to encounter recall issues.

Our data indicate that for caregivers, compliance to dose is less challenging than continuation of zinc for the advised number of days, which is made difficult by the tendency to terminate treatment once a child appears to have recovered from diarrhea. Since the majority of diarrheal episodes among children under–five are acute (ie, typically 3–7 days), there is considerable discordance between the duration of illness and the advised therapeutic course. In a recently published RCT, the majority of participants (73.7%) could not be included in per–protocol analyses because treatment of both placebo– and zinc–randomized children was discontinued when diarrhea halted [9]. In order to increase the proportion of children receiving the full zinc treatment regimen, future programs must address the inclination of caregivers to terminate treatment once a child appears to have recovered. To this end, messages disseminated in the community should emphasize the general health benefits of zinc that extend beyond diarrhea treatment. In addition, it is important that providers are trained to not only counsel caregivers on the appropriate duration of supplementation but to also explain the rationale for continuing zinc after diarrheal symptoms have subsided. Formative research should be conducted to identify salient messages that promote the use of zinc during both diarrhea and convalescence [6,19]. These strategies should succeed in increasing the proportions of caregivers who prefer zinc for diarrhea treatment and perceive zinc as beneficial to a child’s overall health.

We observed a strong correlation between receipt of proper zinc treatment instructions and the odds a child received the age appropriate dose for 14 days. This finding is critical as it underscores the willingness of caregivers to heed the diarrhea treatment advice of providers and thus the importance of ensuring providers are well–trained. We did not find any evidence of children receiving more than one zinc dose per day, but six infants 2–6 months of age received the dose intended for older children. Future diarrhea treatment programs should ensure adequate training of providers in the complex zinc dosage guidelines, especially for young infants. Providers might benefit from visual demonstrations and hands–on practice in the preparation of each of the four zinc syrup/tablet doses. This approach could potentially maximize providers’ retention of age cut–offs and ability to communicate such information to caregivers.

Our findings confirm reports that zinc treatment does not interfere with adherence to ORS [6,8], as all caregivers who reported being instructed to administer ORS complied. Future programs should continue simultaneous scale–up of ORS and zinc in rural India. Moreover, provider trainings should emphasize the importance of advising both products while program implementers concentrate on ensuring supply chain sustainability and prevention of stock–outs.

This study is limited by the reliance on caregiver report to gauge provider instructions on zinc treatment dose and duration. We reduced the threat of recall bias among caregivers by restricting inclusion to children with diarrhea occurring in the last 7 days who were treated with zinc in the 3 days preceding the initial household visit. Possession of zinc products and packaging among all enrolled caregivers at the first household visit suggests that zinc procurement and thus receipt of zinc instructions occurred recently relative to the timing of the visit, thereby lessening the likelihood of misreporting. To prevent recall issues at follow–up, we employed tracking forms to assist caregivers in monitoring treatment of the episode.

This study is also limited by an inability to stratify estimates of adherence by the sector or specific provider cadre from which zinc was procured, since the overwhelming majority of zinc was advised by ASHAs (85.8%). This limitation highlights larger programmatic questions concerning the limited availability of zinc stocks and the low propensity to advise zinc treatment in the private–sector and through certain public–sector outlets [15]. Nonetheless, our findings add to the evidence base on zinc adherence and suggest that among children prescribed zinc within select DAZT program areas, the quality of zinc treatment is generally high albeit with room for improvement.
